# Transient Receptor Potential Vanilloid 1 Function at Central Synapses in Health and Disease

**DOI:** 10.3389/fncel.2022.864828

**Published:** 2022-04-18

**Authors:** Rodrigo C. Meza, Carlos Ancatén-González, Chiayu Q. Chiu, Andrés E. Chávez

**Affiliations:** ^1^Centro Interdisciplinario de Neurociencia de Valparaíso (CINV), Instituto de Neurociencias, Universidad de Valparaíso, Valparaíso, Chile; ^2^Programa de Doctorado en Ciencias, Mención Neurociencia, Universidad de Valparaíso, Valparaíso, Chile

**Keywords:** vanilloid receptor, endocannabinoids, synaptic function, brain, neurotransmision

## Abstract

The transient receptor potential vanilloid 1 (TRPV1), a ligand-gated nonselective cation channel, is well known for mediating heat and pain sensation in the periphery. Increasing evidence suggests that TRPV1 is also expressed at various central synapses, where it plays a role in different types of activity-dependent synaptic changes. Although its precise localizations remain a matter of debate, TRPV1 has been shown to modulate both neurotransmitter release at presynaptic terminals and synaptic efficacy in postsynaptic compartments. In addition to being required in these forms of synaptic plasticity, TRPV1 can also modify the inducibility of other types of plasticity. Here, we highlight current evidence of the potential roles for TRPV1 in regulating synaptic function in various brain regions, with an emphasis on principal mechanisms underlying TRPV1-mediated synaptic plasticity and metaplasticity. Finally, we discuss the putative contributions of TRPV1 in diverse brain disorders in order to expedite the development of next-generation therapeutic treatments.

## Introduction

TRPV1 is a nonselective ligand-gated cation channel predominantly expressed in peripheral endings of small diameter nociceptive primary afferent neurons, where its activation is associated with nociception and pain sensation including thermal hyperalgesia (Caterina et al., 2000; Davis et al., 2000; Brederson et al., 2013). This homotetrameric receptor can be activated by a wide range of stimuli, including heat, changes in pH, exogenous compounds such as capsaicin (Caterina et al., 1997) and endogenous lipid ligands including endovanilloids/endocannabinoids such as anandamide (AEA) (Ross, 2003; Toth et al., 2009). Also, TRPV1 channels are located on the central endings of nociceptive neurons in the spinal cord that make synaptic contact with second order neurons (Caterina et al., 1997; Hwang et al., 2004). Pharmacological activation of presynaptic TRPV1 channels modulates glutamatergic transmission by increasing the frequency of miniature excitatory postsynaptic currents (mEPSCs) and by inhibiting evoked EPSCs (Tognetto et al., 2001; Baccei et al., 2003; Medvedeva et al., 2008).

Although first identified and cloned in peripheral afferent fibers (Caterina et al., 1997), accumulating evidence using diverse experimental approaches indicates that TRPV1 is also expressed in different brain regions including prefrontal cortex, amygdala, brainstem, hypothalamus, cerebellum and the hippocampal formation (Sasamura et al., 1998; Mezey et al., 2000; Sanchez et al., 2001; Roberts et al., 2004; Toth et al., 2005; Cristino et al., 2006; Starowicz et al., 2008; Kauer and Gibson, 2009; Huang et al., 2014; Kumar et al., 2018). While brain TRPV1 is seemingly expressed at lower levels than the peripheral counterparts (Cavanaugh et al., 2011), its activation has been linked to different forms of synaptic plasticity and metaplasticity. Moreover, TRPV1 likely modulates anxiety, fear and panic responses *in vivo* (Almeida-Santos et al., 2013; Aguiar et al., 2014; Terzian et al., 2014) and also the severity of neurological and cognitive disorders, including epilepsy, depression and Alzheimer’s Disease (Chen et al., 2017; Balleza-Tapia et al., 2018; Wang et al., 2019, 2020a; Du et al., 2020). However, the mechanisms by which brain TRPV1 regulates synaptic function and behavior remains far from being completely understood.

## Presynaptic Transient Receptor Potential Vanilloid 1 Channels in the Brain

Capsaicin, the pungent ingredient in hot chili peppers, acts as an exogenous TRPV1 agonist, and it has been shown to enhance transmitter release in the spinal cord (Gamse et al., 1979; Baccei et al., 2003; Kim et al., 2009), supporting the presence of TRPV1 on presynaptic nerve terminals (Tominaga et al., 1998; Cortright et al., 2007). Consistent with this idea, TRPV1 has also been reported at excitatory presynaptic terminals in various brain regions, including the striatum, substantia nigra, hypothalamus, hippocampus, dorsolateral periaqueductal gray and medial prefrontal cortex (Karlsson et al., 2005; Xing and Li, 2007; Musella et al., 2009; Anstotz et al., 2018; Zhang et al., 2020). In these regions, its pharmacological activation facilitated spontaneous excitatory postsynaptic current (EPSC) frequency but not amplitude, consistent with a presynaptic site of action. In the hypothalamus, hippocampus and entorhinal cortex, TRPV1 activation also increased the frequency of spontaneous inhibitory postsynaptic currents (IPSCs; Banke, 2016). These results support a presynaptic localization of TRPV1 channels in glutamatergic and γ-aminobutyric acid (GABA) ergic nerve terminals, whose activation presumably increases intracellular calcium, resulting in enhanced synaptic transmission.

**FIGURE 1:**
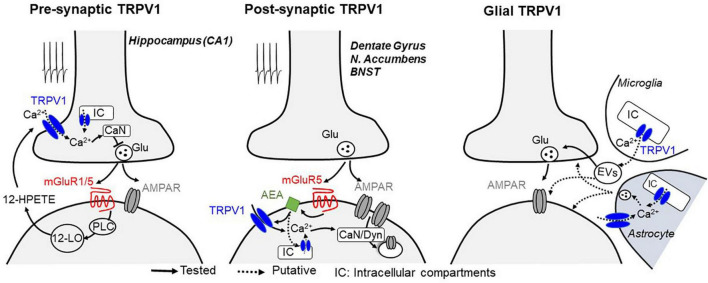
Mechanisms described for TRPV1-mediated plasticity at central synapses. Glutamate release during synaptic stimulation activates Gq-coupled mGluRs, leading to the activation of phospholipase C (PLC) and downstream calcium-regulated enzymes, which likely triggers the biosynthesis of lipids including 12-HPETE (presynaptic TRPV1-LTD, *left*) or AEA (postsynaptic TRPV1-LTD, *middle*), two endocannabinoid/endovanilloid known to activate TRPV1 receptors. At presynaptic terminals, activation of TRPV1 on plasma membrane or intracellular compartments (IC) leads to a calcium (Ca^2+^)-dependent long-term depression of synaptic vesicle release, whereas when located at postsynaptic compartments, TRPV1 activation promotes a long-lasting, clathrin- and dynamin (Dyn)-dependent endocytosis of AMPA receptors. Notably, stimulation of calcium-sensitive phosphatase calcineurin (CaN) resulting from TRPV1 activation is likely to be a common mechanism to promote both pre and postsynaptic long-term changes of synaptic efficacy. TRPV1 channels are also expressed in glial cells (*right*), where its activation can modulate synaptic efficacy presumably through the production and release of inflammatory mediators through microglia-derived extracellular vesicles (EVs) or by Ca^2+^-dependent release of gliotransmitters from astrocytes.

Although seemingly contradictory, TRPV1 activation has also been shown to reduce the release of GABA and glutamate (Gibson et al., 2008; Lu et al., 2017; Zhang et al., 2020). In the hippocampus, TRPV1 mediates a form of long-term depression (LTD) at excitatory inputs onto GABAergic interneurons in the CA1 area of the hippocampus (Gibson et al., 2008). Such synaptic plasticity is dependent on presynaptic TRPV1 that are activated by the postsynaptically produced arachidonic acid metabolite 12-HPETE. At the presynaptic site, TRPV1 opening likely permeates calcium and subsequent activation of calcium-sensitive pathways to persistently depress transmitter release (Jensen and Edwards, 2012; Figure 1). It is noteworthy that a similar form of presynaptic LTD, involving a TRPV-like channel, has also been described in invertebrates (Yuan and Burrell, 2010, 2013), suggesting evolutionary conservation of this synaptic regulatory mechanism.

## Postsynaptic Transient Receptor Potential Vanilloid 1 Channels in the Brain

TRPV1 also mediates postsynaptic forms of LTD (Chavez et al., 2010; Grueter et al., 2010; Puente et al., 2011), strongly suggesting that these channels are also present postsynaptically. In the dentate gyrus and nucleus accumbens, repetitive stimulation of glutamatergic afferents triggers TRPV1-mediated LTD of excitatory synaptic efficacy through a mechanism that involves metabotropic glutamate receptor (mGluR) signaling and clathrin-dependent endocytosis of the α-amino-3-hydroxyl-5-methyl-4-isoxazole-propionate-type glutamate receptors (AMPARs; Chavez et al., 2010; Grueter et al., 2010). In the nucleus accumbens, mild pharmacological activation of muscarinic M1 receptors can also trigger persistent excitatory depression that requires TRPV1 (Neuhofer et al., 2018). It remains to be determined whether M1 receptors can play a role in activity-dependent TRPV1-mediated LTD. Interestingly, in the extended amygdala, TRPV1-mediated LTD selectively required mGluR5 but not mGluR1 (Puente et al., 2011), suggesting that not all G-protein coupled receptors can equally activate TRPV1 at synapses. Notably, TRPV1 activation also induces clathrin-dependent internalization of type A GABA receptors (GABA_A_Rs), showing a preference for somatic over dendritic compartments of dentate granule cells (Chavez et al., 2014). Because TRPV1 may be expressed in distinct intracellular compartments (Gallego-Sandin et al., 2009; Dong et al., 2010; Samie and Xu, 2011), it is possible that this synapse-specificity of TRPV1-mediated suppression of inhibition arise from channel expression in specific subcellular domains. Moreover, it remains unclear how TRPV1 can be endogenously activated to modulate GABAergic synaptic plasticity.

Although diverse forms of TRPV1-mediated LTD can be induced in the hippocampus (Table 1, i.e., CA1 vs dentate gyrus), TRPV1 channels themselves may be acting in the same way (Figure 1). For instance, calcium influx may be triggered by activation of either pre- or postsynaptic TRPV1, resulting in the recruitment of calcium-sensitive enzymes such as calcineurin (also known as protein phosphatase 2B), the predominant calcium/calmodulin-dependent serine/threonine phosphatase that maintains the appropriate phosphorylation status of many ion channels present at presynaptic and postsynaptic sites (Huang et al., 2021). Indeed, several forms of pre and postsynaptic forms of TRPV1-mediated LTD require calcineurin (Gibson et al., 2008; Chavez et al., 2010; Grueter et al., 2010; Yuan and Burrell, 2010, 2013; Jensen and Edwards, 2012). Thus, it is possible that TRPV1 may initiate an important negative feedback via calcineurin in response to increased neuronal activity. Further investigations are required to determine whether calcineurin is the main actor in TRPV1-mediated LTD. The presence of multiple phosphorylation sites in TRPV1 implies possible regulatory actions by different kinases, including calcium calmodulin, protein kinase A and C (Rosenbaum et al., 2004; Rosenbaum and Simon, 2007) as well as Src kinase (Jin et al., 2004). Their contribution to TRPV1-mediated plasticity remains unclear.

**TABLE 1:** TRPV1-mediated synaptic plasticity in the mammalian brain.

Brain area	Synapse type	Synaptic plasticity	Induction protocol	Expression mechanism	Endogenous agonist	References
Hippocampus	Excitatory inputs to GABAergic interneurons	LTD	HFS	Presynaptic Ca^2+^ Calcineurin	Endovanilloid 12-HPETE	Gibson et al., 2008 Jensen and Edwards, 2012
Dentate gyrus	medial perforant path to dentate granule cells excitatory inputs	LTD	1Hz Paired Protocol	Postsynaptic Ca^2+^ Calcineurin AMPA receptor endocytosis	Anandamide	Chavez et al., 2010
Nucleus accumbens	Excitatory inputs to D2+ neurons	LTD	LFS	Postsynaptic AMPA receptor endocytosis	Anandamide	Grueter et al., 2010
Amygdala, stria terminalis	Excitatory inputs	LTD	LFS	Postsynaptic	Anandamide	Puente et al., 2011
Superior colliculus	Excitatory inputs*	LTD	HFS	Presynaptic?	Endovanilloid 12-HPETE	Maione et al., 2009

LFS, low frequency stimulation; HFS, high frequency stimulation; LTD, long-term depression. *Transiently in juvenile but not in adult.

## Glial Transient Receptor Potential Vanilloid 1 Channels in the Brain

Modulation of synaptic transmission could also be the result of activation of TRPV1 in glia (Figure 1). For instance, proinflammatory molecules such as the bioactive phospholipid lysophosphatidic acid has been shown to activate TRPV1 by directly binding to its C-terminal (Nieto-Posadas et al., 2011). While microglial TRPV1 is primarily localized in mitochondrial but not plasma membranes (Miyake et al., 2015), its activation indirectly enhances glutamatergic transmission in cortical neurons (Marrone et al., 2017), presumably through the production and release of inflammatory mediators (Miyake et al., 2015; Marrone et al., 2017; Kong et al., 2019). TRPV1 may also be expressed in astrocytes (Toth et al., 2005; Marinelli et al., 2007), although most of the recent evidence suggests that this is induced in response to brain injury. For example, TRPV1 activation in substantia nigral astrocytes produces the ciliary neurotrophic factor, which prevents the active degeneration of dopaminergic neurons in rat models of Parkinson’s disease (Nam et al., 2015). Hypoxic ischemia also reportedly triggers the expression of astrocytic TRPV1 and consequent release of pro-inflammatory cytokines from astrocytes to neighboring neurons in epileptogenesis (Wang et al., 2019). However, whether TRPV1 in astrocytes can modify synaptic function and plasticity in neurons under physiological conditions remains unclear. It is also important to note that capsaicin has been reported to exert direct effects on voltage-sensitive ion channels (Hagenacker et al., 2011; Yang et al., 2014; Pasierski and Szulczyk, 2020) and thus may modulate neurotransmitter release in a TRPV1-independent manner in cases where TRPV1 antagonists or knockouts were not assessed. For example, in the lateral amygdala (LA), capsaicin reportedly modifies LTD through activation of TRPM1 (Gebhardt et al., 2016). Last but not least, activation of postsynaptic TRPV1 may induce the production of calcium dependent retrograde signaling molecules to suppress neurotransmitter release (see below).

## Interaction Between Transient Receptor Potential Vanilloid 1 and the Endocannabinoid System

Within the hippocampus, inhibition of TRPV1 dramatically reduces glutamatergic input to oriens-lacunosum-moleculare (OLM) interneurons (Hurtado-Zavala et al., 2017) and can block presynaptic LTD of excitatory synapses at inhibitory GABAergic interneurons but not pyramidal neurons (Gibson et al., 2008). Notably, this TRPV1-dependent presynaptic modulation of synaptic plasticity is similar in many ways to that reported for endocannabinoid (eCB)-mediated LTD that involves the activation of presynaptic type 1 cannabinoid (CB1) receptors (Castillo et al., 2012; Katona and Freund, 2012). For instance, in both forms of LTD, postsynaptic mGluRs are involved in promoting the production of a lipid messenger that then acts retrogradely to suppress transmitter release in a long-term manner. In postsynaptic TRPV1-LTD, mGluR activation promotes the production of anandamide (AEA), a major endocannabinoid (Chavez et al., 2010; Grueter et al., 2010; Puente et al., 2011). In this case, AEA likely acts directly on postsynaptic TRPV1 to induce endocytosis of AMPARs expressed on dendritic spines, reducing their number and consequently the long-term reduction in excitatory synaptic strength. Given the ability of eCBs like AEA to activate TRPV1, it has been suggested that TRPV1 may act as an ionotropic eCB receptor (De Petrocellis et al., 2017; Muller et al., 2018; Storozhuk and Zholos, 2018) with central neuromodulatory effects that either mimic or oppose those exerted by CB1 receptors.

The factors that determine whether eCBs act on TRPV1 or CB1 receptors to regulate synaptic transmission remain unclear. Interestingly, in the bed nucleus of stria terminalis, L-type calcium channel activation in dendrites leads to production and secretion of 2-arachidonoylglycerol (2-AG) that acts on presynaptic CB1 receptors in short-term depression, whereas AEA secretion following mGluR5 activation acts on postsynaptic TRPV1 in LTD (Puente et al., 2011). Additionally, other eCBs such as N-arachidonoyl-dopamine (Huang et al., 2002), can also activate both TRPV1 and CB1R, producing opposing actions on synaptic transmission (Marinelli et al., 2007). Moreover, it has been proposed that postsynaptic TRPV1 activation by AEA can regulate the magnitude of 2-AG-mediated tonic inhibition of perisomatic GABAergic transmission in CA1 pyramidal neurons (Lee et al., 2015), putatively through TRPV1-induced reduction of 2-AG levels (Maccarrone et al., 2008).

Spike-timing-dependent long-term potentiation (tLTP) in the striatum and neocortex, important for acquiring new associative memories, requires both TRPV1 and CB1 receptors (Cui et al., 2015, 2018). Notably, capsaicin activation of TRPV1 alone in the neocortex suppressed excitatory transmission (Cui et al., 2018). The mechanisms underlying TRPV1 and CB1 receptor co-dependency and their conversion from depressive to potentiating effects remain to be investigated. The signaling pathways downstream of TRPV1 and CB1 receptor activation may be dynamically inter-regulated. Indeed, the lack of TRPV1 reportedly modifies the expression and localization of different components of the eCB system, causing a shift from CB1 receptor-mediated LTD to LTP in the dentate gyrus (Egana-Huguet et al., 2021). While these results suggest an ability of eCBs to act through both CB1 and TRPV1 receptors, the factors that determine directionality of synaptic plasticity and functional consequences in behavior need to be determined.

## Transient Receptor Potential Vanilloid 1 in Metaplasticity

**FIGURE 2:**
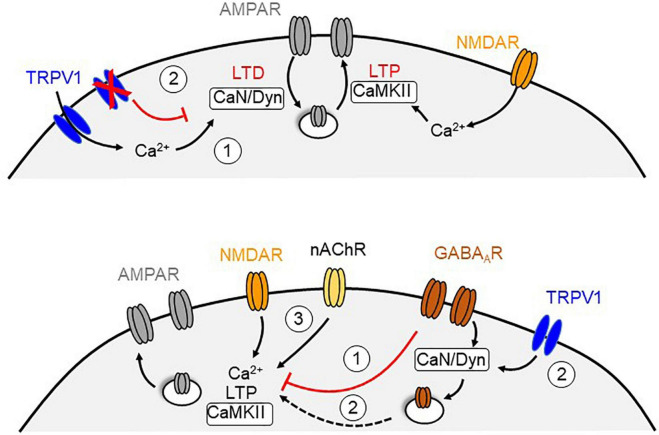
Potential mechanisms for TRPV1-dependent metaplasticity at central synapses. *Top*, Calcium influx through TRPV1 channels triggers a form of postsynaptic LTD that require endocytosis of AMPARs, which may counterbalance the level of N-methyl-D-aspartate receptor (NMDAR) dependent LTP (1). In contrast, blockade or genetic deletion of TRPV1 channels recovers NMDAR-mediated LTP that may be due to the absence of TRPV1-LTD (2). *Bottom*, NMDAR-dependent LTP can be reduced by activation of GABA_A_Rs (1). Activation of TRPV1 channels could triggers a calcineurin (CaN) and dynamin (Dyn)-dependent endocytosis of GABA_A_Rs that remove the inhibitory suppression of the LTP (2). Exogenous activation of nicotinic acetylcholine receptors (nAChR) may oppose the influence of GABAergic inhibition and rescue LTP in the absence of TRPV1 likely by increasing calcium levels (3).

TRPV1 activity can alter the inducibility of long-term synaptic plasticity (Table 2). In the entorhinal cortex, LTP does not occur unless TRPV1 is blocked (Banke, 2016). Similarly, activation of TRPV1 with capsaicin attenuates the magnitude of LTP in the LA (Zschenderlein et al., 2011). Given the ability of TRPV1 activation to induce endocytosis of AMPARs, it is possible that the simultaneous expression of TRPV1-mediated LTD occludes or opposes LTP under these conditions (Figure 2, top). In contrast, TRPV1 knockout mice show deficits in LTP at the Schaffer collateral–commissural pathway to CA1 hippocampal neurons (Marsch et al., 2007), which can be rescued by activating OLM neurons with nicotine (Hurtado-Zavala et al., 2017). Moreover, these TRPV1 deficient mice reportedly show impaired hippocampal-dependent learning, fear conditioning and anxiety, effects that cannot be explained by alterations in nociception. Additionally, capsaicin exposure in wildtype mice enhances CA1 LTP and spatial memory (Li et al., 2008; Bennion et al., 2011), potentially by decreasing inhibition via TRPV1-mediated iLTD (Figure 2, bottom). Hippocampal TRPV1 activation also enables spatial memory retrieval under stressful conditions and rescues LTP in slices derived from swim-stressed mice (Kulisch and Albrecht, 2013). Although these findings support a role for brain TRPV1 in regulating synaptic function and behavior, it remains unclear how TRPV1 is able to bidirectionally modulate metaplasticity.

**TABLE 2:** TRPV1 metaplasticity in the mammalian brain.

Brain area	Synapse type	Effects	Induction protocol	References
Hippocampus	Schaffer collateral to CA1 Schaffer collateral to CA1 Schaffer collateral to CA1	Reduced LTP Enhanced LTP and reduced LTD Enhanced LTP	HFS HFS-LFS TBS-HFS	Marsch et al., 2007 Li et al., 2008 Bennion et al., 2011
Entorhinal cortex	Excitatory Inputs to layer II/III	TRPV1 blockade enables LTP	HFS	Banke, 2016
Lateral amygdala	Cortical Excitatory inputs	Depressed LTP in ether Enhanced LTP in isoflurane Depressed LTP in ether Blocked stress-induced impairment of LTP	HFS HFS	Zschenderlein et al., 2011 Kulisch and Albrecht, 2013
Dorsolateral striatum	Excitatory inputs	Co-dependent CB1-TRPV1 LTP	STDP	Cui et al., 2015
Neocortex	Excitatory inputs	Co-dependent CB1-TRPV1 LTP	STDP	Cui et al., 2018

*LFS, low frequency stimulation; HFS, high frequency stimulation; TBS, theta-burst stimulation; STDP, spike-timing dependent plasticity; LTP, long-term potentiation; LTD, long-term depression.*

## Transient Receptor Potential Vanilloid 1 Might Regulate Hippocampal Development/Circuit Maturation

In addition to regulating synaptic function, a role for TRPV1 in development is indicated by the ability of capsaicin to activate these channels in hippocampal Cajal-Retzius cells (Anstotz et al., 2018). These cells are a major source of the extracellular matrix protein reelin, which is essential for development (Del Rio et al., 1997; Frotscher, 1997). Indeed, Cajal-Retzius cells can powerfully excite GABAergic interneurons of the molecular layer in the dentate gyrus via TRPV1 activation (Anstotz et al., 2018), supporting the idea that TRPV1 may shape layer-specific interneuron connectivity in hippocampal development. Interestingly, the density of Cajal-Retzius cells decreases during postnatal development, raising that possibility that functional TRPV1 receptors may drive this progressive reduction by calcium-dependent apoptosis. Consistent with this idea, in the mouse hippocampus, it has been suggested that the expression of both the messenger (mRNA) and the protein are low in the early stages (<P15), increasing from the eighth postnatal week and then decreasing towards postnatal week 16 (Huang et al., 2014). Whether changes in the expression and activation of TRPV1 in early stages of development (P < 20) play a role in circuit maturation remain unclear. Notably, TRPV1 is transiently expressed in retinal afferents to principal neurons in the superior colliculus, where excitatory synapses onto them show a form of activity-dependent LTD that is dependent on TRPV1 in juvenile but not adult animals (Maione et al., 2009).

## Transient Receptor Potential Vanilloid 1 in Mental Disorders and Neurological Diseases

TRPV1 has been linked to numerous neurological and neuropsychiatric disorders, with channel desensitization and blockade as primary therapeutic objectives (Edwards, 2014; Weng et al., 2015; Singh et al., 2019; Escelsior et al., 2020; Wang et al., 2020b; Allain et al., 2021; Asth et al., 2021; Zhou et al., 2021; Akhilesh et al., 2022). Emerging evidence indicates that TRPV1 expression is increased following diverse forms of stress (Li et al., 2008; Rubino et al., 2008; Kulisch and Albrecht, 2013; Saffarzadeh et al., 2015; Garami et al., 2020; Aghazadeh et al., 2021) to likely drive maladaptive responses. For example, chronic exposition to heat can upregulate TRPV1 channels in the brain, probably by the generation of reactive oxygen species (Aghazadeh et al., 2021), but the functional effect of this expression in regulating neuronal function has not been studied. Interestingly, TRPV1 antagonists have been shown to induce hyperthermia in human clinical trials (Garami et al., 2020), suggesting that TRPV1 channels may play a role in regulating core body temperature. The central mechanisms are unknown but may involve TRPV1 channels in the hypothalamus (Iida et al., 2005; Sharif-Naeini et al., 2008; Sudbury and Bourque, 2013; Zaelzer et al., 2015; Molinas et al., 2019). Notably, TRPV1 blockers act through different mechanisms depending on animal model, specifically targeting proton-activated TRPV1 domains in rodents, but heat and proton-activated TRPV1 regions in humans (Garami et al., 2020). In neuropathic pain, nerve damage alters synaptic circuits in the spinal cord, brainstem and cortex, involving neurons and glial cells, that leads to persistent amplification of pain signals (Giordano et al., 2012; Tsuda et al., 2017; Arribas-Blazquez et al., 2019). It is unclear whether heightened TRPV1 function in these cell types contribute to such plasticity. However, in support of this idea, activation of microglial TRPV1 enhances glutamatergic neurotransmission and the presence of neuronal TRPV1 increases action potential firing, which could enhance cortical activity and be crucial for the emotional alteration in chronic pain (Marrone et al., 2017).

Moreover, TRPV1 may tonically modulate emotional defensive responses such as anxiety, fear and panic (Millan, 2003; Mcnaughton and Corr, 2004; Terzian et al., 2009; Canteras et al., 2010; Casarotto et al., 2012; Aguiar et al., 2015; Gobira et al., 2017). Stress conditioning is accompanied by augmented TRPV1 levels in both the hippocampus and LA (Kulisch and Albrecht, 2013; Navarria et al., 2014). Intracranial injection of TRPV1 antagonists or its genetic deletion in these limbic brain regions promotes anxiolytic and antidepressant states as well as decrease cue and contextual fear conditioning (Kasckow et al., 2004; Marsch et al., 2007; Rubino et al., 2008; Aguiar et al., 2009; Terzian et al., 2014; Wang et al., 2017). It has been proposed that blocking TRPV1 may be antidepressive by consequently increasing serotonin levels (Manna and Umathe, 2012). On the other hand, some reports indicate that activation of TRPV1 rescued stress-induced impairment of hippocampal LTP and spatial memory retrieval (Li et al., 2008). In contrast, activation of TRPV1 in control conditions suppresses LTP in LA by modulation of the nitric oxide system (Zschenderlein et al., 2011). More work is needed to understand how the blockade or deletion of TRPV1 promotes anxiolytic and anti-depressive states, while its overexpression rescues cognitive deficiencies caused by stress.

Interestingly, TRPV1 expression is reduced in a transgenic model of Alzheimer’s Disease (AD; Du et al., 2020), which is a neurodegenerative disorder characterized by memory and learning deficits. Disrupted TRPV1-mediated synaptic plasticity and metaplasticity may be involved, but this remains a matter of debate. For instance, TRPV1 activation or reinsertion prevents the shift in excitatory/inhibitory balance and restores normal brain oscillatory activity (Balleza-Tapia et al., 2018), rescues hippocampal LTP and improves memory performance (Chen et al., 2017; Du et al., 2020; Wang et al., 2020a,^2021^). However, a recent report suggests that the absence of TRPV1 rescues memory deficits in AD (Kim et al., 2020). The diverse influences of TRPV1 channels in pathological condition may reflect cause or consequence of brain disorders. More time-lapse studies are needed to fully understand the exact contribution of TRPV1 in disease etiology and pathophysiology.

## Conclusion and Future Directions

Accumulating evidence indicate that, in addition to its well-known role in modulating pain transduction, TRPV1 seems to play an important role in regulating brain synaptic transmission and plasticity. TRPV1 can be expressed both presynaptically and postsynaptically, and depending on the specific synapse, it can either increase or decrease neurotransmission. The impact of glial TRPV1 function on central synapses requires further investigation. Given the ubiquitous expression of TRPV1 throughout the brain, it is not surprising that TRPV1 has been implicated in several neurological and psychiatric disorders such as epilepsy, anxiety, and depression as well as drug-addiction disorders (Edwards, 2014; Singh et al., 2019; Escelsior et al., 2020; Allain et al., 2021; Asth et al., 2021; Zhou et al., 2021). Future work will expand our appreciation of the role and the exact mechanism underlying TRPV1-mediated changes in synaptic transmission and plasticity at central synapses in health and disease. For example, it is unknown whether TRPV1-mediated plasticity is present at GABAergic synapses and how it influences circuit function. It is also unclear under what conditions AEA acts on TRPV1 or CB1 receptors to modify synaptic function and behavior. Whether glial TRPV1 can be activated by AEA or other vanilloids is unclear as well. Moreover, different neuromodulators including serotonin, histamine, or prostaglandins are known to stimulate TRPV1 in the periphery (Cesare et al., 1999; Premkumar and Ahern, 2000; Vellani et al., 2001). Whether neuromodulation regulates brain function and behavior in a TRPV1-dependent manner remains unknown.

## Author Contributions

AEC and CQC conceived of the general ideas presented in this review and supervised the work. CA-G and RM contributed equally to all sections and helped with figure and table construction. All authors made significant contributions to the information content and writing of this final manuscript.

## Conflict of Interest

The authors declare that the research was conducted in the absence of any commercial or financial relationships that could be construed as a potential conflict of interest.

## Publisher’s Note

All claims expressed in this article are solely those of the authors and do not necessarily represent those of their affiliated organizations, or those of the publisher, the editors and the reviewers. Any product that may be evaluated in this article, or claim that may be made by its manufacturer, is not guaranteed or endorsed by the publisher.
